# Noncommunicable diseases, injuries, and mental health: the triple burden in Africa

**DOI:** 10.11604/pamj.2022.43.167.38392

**Published:** 2022-12-02

**Authors:** Mary Amuyunzu-Nyamongo

**Affiliations:** 1 African Institute for Health and Development (AIHD), Nairobi, Kenya

**Keywords:** Noncommunicable diseases, injuries, mental health, Africa

## Editorial

Cardiovascular diseases, cancers, chronic respiratory conditions, diabetes, mental and neurological disorders are the key noncommunicable diseases (NCDs) responsible for high morbidity and mortality in sub-Saharan Africa (SSA) [1]. The main factors driving these conditions include weaknesses in the implementation of critical control measures including prevention, diagnosis, management, and palliative care at all levels of the health system. The achievement of the global target of the *"reduction of premature mortality from four main NCDs (cardiovascular disease (CVD), diabetes, hypertension and chronic respiratory infections) by 25% from 2010 levels by 2025"* will depend on achieving the key risk factor targets for NCDs (tobacco and alcohol use, salt intake, obesity, and raised blood pressure and glucose) [2]. However, it is clear that most countries, and more specifically those in Africa, are far from attaining this target [3]. The Lancet NCDs and Injuries (NCDI) Poverty Commission found that NCDs and injuries cause over a third of all annual deaths among the poorest a billion, killing 800,000 annually among those under the age of 40 [4]. The report noted (in this group) NCDs and injuries kill more people every year than HIV/AIDS, tuberculosis (TB), and maternal deaths combined. The cost for managing these conditions is too high for the poorest a billion, with between 19 and 50 million people every year spending catastrophic amounts of money in out-of-pocket payments [5].

Mental health, which is closely associated with cancer, diabetes, cardiovascular, respiratory diseases and other NCDs is a major concern in the region [6]. Mental health conditions including anxiety, depression, psychosis, neurological and substance use disorders, account for about 25% of all non-fatal disease burden. More than 700,000 people die yearly because of suicide. This is happening within a context of few trained mental healthcare professionals at all levels of the health system. It is notable that there can be no health without mental health. Indeed, between 2010 and 2050, the burden of mental and substance use disorders in sub-Saharan Africa is expected to increase by 130%, and 216.000 extra full time mental health staff will be required for optimal care [7]. Examining these three conditions - NCDs, mental health and injuries - shows that the African continent has a triple burden. The rise of NCDs, injuries and mental health conditions is estimated to cause more premature deaths on the continent than all other conditions combined by 2030 and, by far, will cause the most deaths and disability by 2063 [6].

**The need for an integrated approach:** there are common underlying conditions (e.g. poverty and unhealthy environments), and commonalities across disease groups in causation, co-morbidity, and care needs of the three conditions. Oftentimes, NCDs co-exist in the same individual, and having one condition increases the risk or impact of the other. The common elements among the three conditions include access to care, social issues, multi-stakeholder engagement, and policy contexts.

**Access to care:** the care requirements are high and costly. Access to medicines is limited and most of the treatment requirements are not covered in the essential medicine list or under universal health coverage. Associated gaps include workforce skills, health financing and access.

**Social issues:** lifestyles are a key driver of NCDs. The consumption of sugar, salt, fatty foods, alcohol, and tobacco are key risk factors with socio-cultural underpinnings. Lack of physical activity is a risk linked to lifestyle but also associated with other sectors including transport, security, and education. Stigma continues to be a key hindrance to accessing care and limits coping strategies.

**Multi-stakeholder engagement:** actions to address these conditions require multi-stakeholder engagement since they go beyond the health sector to involve trade, security, agriculture (food and nutrition), education, environment, transport, etc. The key challenge has been the inability to rally the other sectors to be fully engaged in NCDs.

**Policy environment:** although countries may have policies to address these conditions, there is a gap in implementation. In addition, some of the mandates are domiciled in different ministries, departments, and agencies. The silo approach to addressing health conditions leads to inadequate attention and duplication of efforts.

**The urgent need for integration of NCDs, injuries and mental health:** the integration of NCDs, mental health and injuries into all levels of healthcare services (particularly at the community level through measures such as responsible task shifting and sharing with community health workers) is critical to the successful management of these conditions. Integration could be at several levels: 1) Service level focus-screening for NCDs and mental health could be done at service points, at first contact with a patient. 2) Health promotion and prevention activities could be implemented to address the three conditions and other health issues. 3) Training of the workforce - re-training and sensitization of health workers on identification, screening, and management. 4) Community health - routine management of these conditions can be effectively done by community health volunteers through effective “task shifting”. 5) Advocacy at the health policy and decision-making levels - most decision makers are directly affected and could be persuaded to put measures in place to address these conditions. 6) Data generation and use is still weak, thereby impacting the ability of the actors to predict and plan appropriately. Data should be disaggregated based on the disease/condition, age, gender, and socio-economic characteristics. 7) Implementation research is key in assessing how best to address these conditions, even within the health and societal constraints that characterize most of the countries in the region. 8) Social mobilization is critical to the design of targeted messaging for specific groups, conditions, and geographic areas. A sensitive social mobilization approach has potential for buy-in by communities and sustainability.
